# Visualizing Polymer Damage Using Hyperspectral Imaging

**DOI:** 10.3390/polym12092071

**Published:** 2020-09-12

**Authors:** Monika Bleszynski, Shaun Mann, Maciej Kumosa

**Affiliations:** 1 NSF I/UCRC for Novel High Voltage/Temperature Materials and Structures at the University of Denver, Denver, CO 80208, USA; mkumosa@du.edu; 2 Tri-State Generation and Transmission Westminster, CO 80234, USA; smann@tristategt.org

**Keywords:** hyperspectral imaging, silicone rubbers, extreme aging, polymer damage, remote sensing

## Abstract

Silicone rubbers (SIRs) are common industrial materials which are often used for electrical insulation including weather sheds on non-ceramic insulators (NCIs). While SIRs are typically resilient to outside environments, aging can damage SIRs’ favorable properties such as hydrophobicity and electrical resistance. Detecting SIR aging and damage, however, can be difficult, especially in service. In this study we used hyperspectral imaging (HSI) and previously investigated aging methods as a proof of concept to show how HSI may be used to detect various types of aging damage in different SIR materials. The spectral signature changes in four different SIRs subjected to four different in-service aging environments all occurred between 400––650 nm. Therefore, remote sensing of NCIs using HSI could concentrate on bands below 700 nm to successfully detect in service SIR damage.

## 1. Introduction

Polydimethylsiloxane (PDMS)-based silicone rubber (SIR) materials are used in a variety of applications, including as industrial sealants, electrical insulation for aircraft, and on high voltage (HV) transmission lines in the form of non-ceramic insulators (NCI) [1]. While SIRs are typically resilient, aging will often reduce their hydrophobic and electrical properties, thus diminishing their mechanical strength and ability to prevent moisture ingress [2,3,4,5,6,7,8,9,10,11,12]. Although current methods exist to determine when damage to SIRs has occurred (i.e., measuring changes in hydrophobicity or material hardness), these testing methods often require specialized or cumbersome equipment [4,5,6,7,8]. Therefore, there is considerable interest in new analysis methods that can identify aging and damage in SIR materials quickly and efficiently.

Hyperspectral imaging (HSI), which utilizes specialized cameras to capture detailed images across multiple wavelengths, could be a potential imaging solution for assessing damage to SIRs [13,14,15,16]. In agriculture, HSI has already shown great promise in detecting diseased crops, such as bark beetle damage in forests, identifying tree species, and even recording the amount of chlorophyll in leaves [17,18,19,20]. Critically, HSI has also been used for identifying constituents and damage in food and animal tissues, and has been successfully used to assess damage to agricultural crops such as corn [21,22,23,24,25]. Furthermore, it has been used for polymeric processing and recycling due to its proven ability to identify differences in materials and structures [21,22,23,24,25], and has been successfully used for detecting, characterizing, and sorting of polymer materials [26,27,28,29]. Thus, it is plausible that HSI technology can be expanded to detect damage in synthetic polymeric materials, and as a result, this technology holds great promise for detecting damage in a variety of materials.

In the field of instrumentation and measurement (IAM), a number of studies have focused on either hyperspectral technology [30,31], HSI for atmospheric and oceanic measurements [32], or chemical detection [33,34]. However, to the authors’ knowledge, no studies within IAM have focused exclusively on HSI and polymer aging. As a result of its potential, we therefore assessed whether HSI can be used to identify material changes in polymeric SIR materials caused by aging.

Room temperature vulcanized (RTV) and high temperature vulcanized (HTV) often refer to formulations or compounding of multiple chemicals into the final material [1,2]. RTV and HTV materials are composed of at least 30 wt % of polydimethylsiloxane (PDMS) [1,2]. NCI HTV insulator SIRs are mixtures of PDMS and of several additives such as fire retardants, fungicides, and fillers to increase stiffness and color [1,2,3,4,5,6,7,8,9,10,11,12,35]. These additives are compounded into the SIR, along with alumina trihydrate, which is the primary fire retardant in NCIs [36]. ATH is added at loadings as high as 66 wt %, and fumed silica filler helps resist aging due to acid rain environments. RTV materials are commonly cured using platinum or tin catalysts, while HTV is the product of a two-component organic peroxide radical cure [1,2,35,36].

SIRs provide the necessary surface hydrophobicity for NCIs, preventing standing water, as rainwater and atmospheric condensation beads on the SIR surface due to its low-energy surface, preventing water ingress [1,2,3,4,5,6,7,8,9,10,11,12]. Excess water on NCI surfaces can cause leakage currents, which can form an electrical path to ground resulting in a local black out or worse [7,8,9,10,11,12]. Oxidative stressors, such as UV and acidic rain, can damage SIRs, decreasing their hydrophobicity [2,3,4,5,6,7,8,9,10,11,12]. Additionally, dry band arcing (DBA) and corona electrical stressors can create nitric acid on the surface, causing further damage [4]. SIRs damaged by acid rain fails sooner under dry band arcing [10], potentially causing catastrophic failure, and it is critical for utilities and transmission/distribution companies to know the condition of their in-service NCIs, especially those exposed to extreme environmental aging conditions. Therefore, we investigated whether hyperspectral imaging be used as a potential polymer analysis and characterization method for experimentally aged polymers, as to the best of our knowledge hyperspectral imaging has not been used to assess aging damage in polymeric materials.

This manuscript is organized as follows: Section 2 describes the details of hyperspectral imaging technology; Section 3 details the materials and methods utilized in this study; Section 4 presents the results and discussion of the findings; and finally, Section 5 presents the conclusions.

## 2. Hyperspectral Imaging

**Figure 1:**
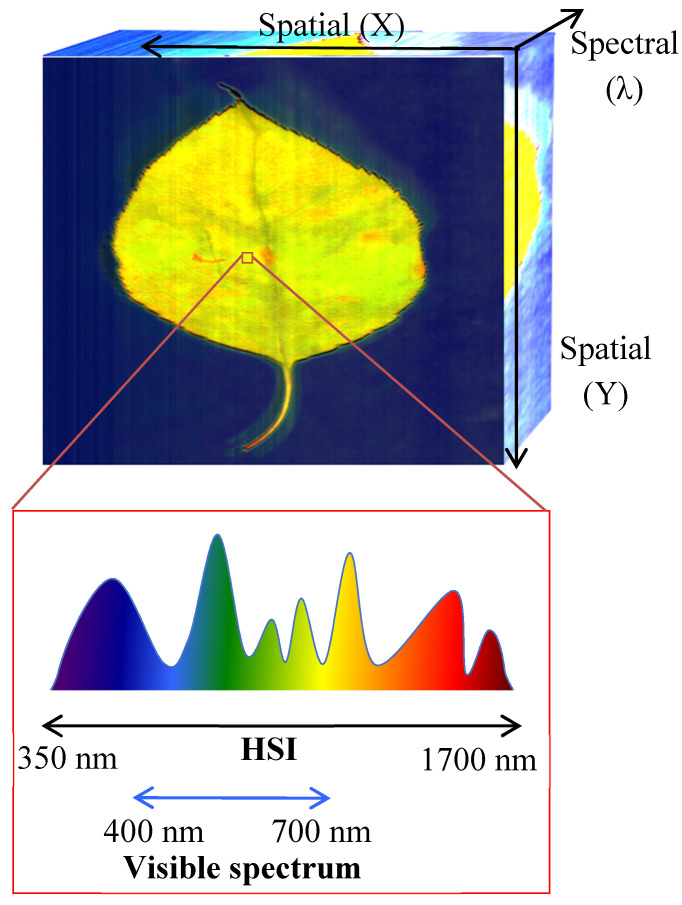
Mockup of a hyperspectral datacube and corresponding wavelength for a particular pixel [13,14,15].

Hyperspectral imaging (HSI) is an emerging technology that uses specialized cameras to divide the light spectrum into multiple wavelength bands [15,16]. Unlike ultraviolet or multispectral cameras, hyperspectral cameras record images using the entire electromagnetic spectrum, which can be used to identify objects or materials [15,16]. While the human eye only sees wavelengths in three bands (red-green-blue) within the visible spectrum (400 to 700 nm), various hyperspectral cameras can capture wavelengths anywhere from 350 to 1700 nm (Figure 1) [14,15,16]. The spectral images obtained using hyperspectral imaging can record hundreds of contiguous spectral channels compared to the spaced 5–10 bands captured by multispectral cameras [16]. This allows for better analysis and visualization of a material or structure.

Another advantage of HSI is in image processing; hyperspectral cameras create 3D images—called hyperspectral cubes—which capture multiple spectral fingerprints in the form of wavelengths for each pixel in an image, assigning different signatures and information such as absorption, reflectance, or fluorescence spectrum data (Figure 1) [13,14,15]. Datacubes have one spectral and two spatial dimensions, which are illustrated in Figure 1 [15]. These datacubes are data rich and can capture more information within a pixel compared to multispectral imaging [15].

Because the shapes of the obtained reflectance spectrums, or spectral signatures, are individually unique, they can be used to identify individual components in an image such as bricks or trees [37]. This information becomes especially valuable when combined with machine learning, especially when enough datasets are provided [23,24,37,38].

When necessary, compact hyperspectral imaging systems can be mounted on unmanned aerial vehicles UAVs for rapid remote sensing of vegetation [17,18,19], as they can reveal much more data about an object than multispectral or infrared cameras. Thus, with just one image, a hyperspectral camera can give detailed information about an object such as chemical composition or plant species, even from a distance [13,14,15,16,17,18,19,20,21,22,23,24,25].

The wavelength band or wavenumber data recorded by HSI cameras can be used to determine the exact composition or characterization of a material, if it can be correlated to a previously known substance [15,16]. By comparison, spectroscopic methods such as Fourier-transform infrared spectroscopy (FTIR) have a range from 4000 to 400 cm^−1^ (2.5 to 25 µm), while 2500 to 780 nm is the typical range for near-infrared spectroscopy (NIRS) [39,40,41]. Thus, the range of the FTIR and NIRS spectrums are well beyond the upper range of HSI, while the lower nm wavelengths are beyond the range of standard FTIR and NIRS (below 700 nm). Using HSI to investigate changes in materials and structures, such as SIRs, could therefore be advantageous for detecting polymer material changes that standard spectroscopic tools cannot identify due to its powerful processing power, speed, and accessibility.

## 3. Materials and Methods

Four different SIR formulations were used in this study, namely: RTV-1 and RTV-2 (room temperature vulcanized one and two component), RTV-2 + 22% aluminum trihydrate (ATH) (Smooth-On, Macungie, PA, USA), and a HTV (high temperature vulcanized) insulator weather-shed. RTV-1 SIRs are commonly used in NCIs as caulking materials near end fittings or between the weather sheds, while weather sheds are made out of RTV-2 SIRs. It should be noted that the RTV-2 with ATH used in this study is our primitive version of a real life HTV used in commercially available NCIs. The RTV-1 and RTV-2 SIRs used in this study were previously investigated in [5,6], respectively. The HTV used in this study came from a virgin commercial NCI from a major international NCI manufacturer (Virginia Beach, VA, USA).

In order to detect changes in SIRs as a result of aging, different types of SIRs were aged in this study and assessed for changes using HSI (Resonon Imaging Systems, Bozeman, MT, USA) under the presumption that even minute changes to the chemical structure of SIR materials due to aging could be detected by HSI. We have previously investigated the aging of RTVs [4,5] and found 0.046% hypochlorous acid (HOCl), UVA, and nitric acid to be effective aging media with noticeable effects including changes in hardness, discolorations, formation of low molecular weight (LMW) oil on the surface, and changes in hydrophobicity [4,5,6], and we utilized these methods in this study (Table 1). We therefore used these previously utilized aging methods in addition to dry band arcing (DBA) to achieve accelerated aging and to induce LMW oil production. A virgin un-aged SIR sample was also included in the first aging study in order to determine differences in wavenumber patterns or characteristics.

Before HSI assessment, each of the four types of samples were aged using various methods, and we have previously utilized some of these methods which are also described in [4,5,6]. Additionally, we have previously found that these aging mechanisms result in noticeable decreases in hydrophobicity and hardness in SIR materials [4,5,6]. Therefore, STRI hydrophobicity and Shore A hardness properties were recorded before and after aging for each sample, and this information is shown in Table 2.

The HTV sample was soaked in commercially produced room temperature 0.046% hypochlorous acid solution for five weeks, after which it was removed from the solution and allowed to dry under ambient room temperature conditions for 24 h before HSI assessment. The RTV-1 sample was placed in a QUV Q-Labs, Inc. Weathering UV chamber for one week at 1.5 W/m^2^ flux, before being removed from the test chamber and assessed using HSI. The LMW oil was left undisturbed on the RTV-1 for HSI analysis. The RTV-2 sample was immersed in an 80 °C nitric acid with a pH of 2.6 for six weeks, before being removed from the solution and allowed to dry ambient room temperature conditions for 24 h before HSI assessment. Finally, the RTV-2 + 22% ATH sample was subjected to dry band arcing according to the ASTM D2303 standard, at 4.5 kVAC for 30 min. The sample material types, aging mechanisms, and aging times are listed in Table 1.

**Figure 2:**
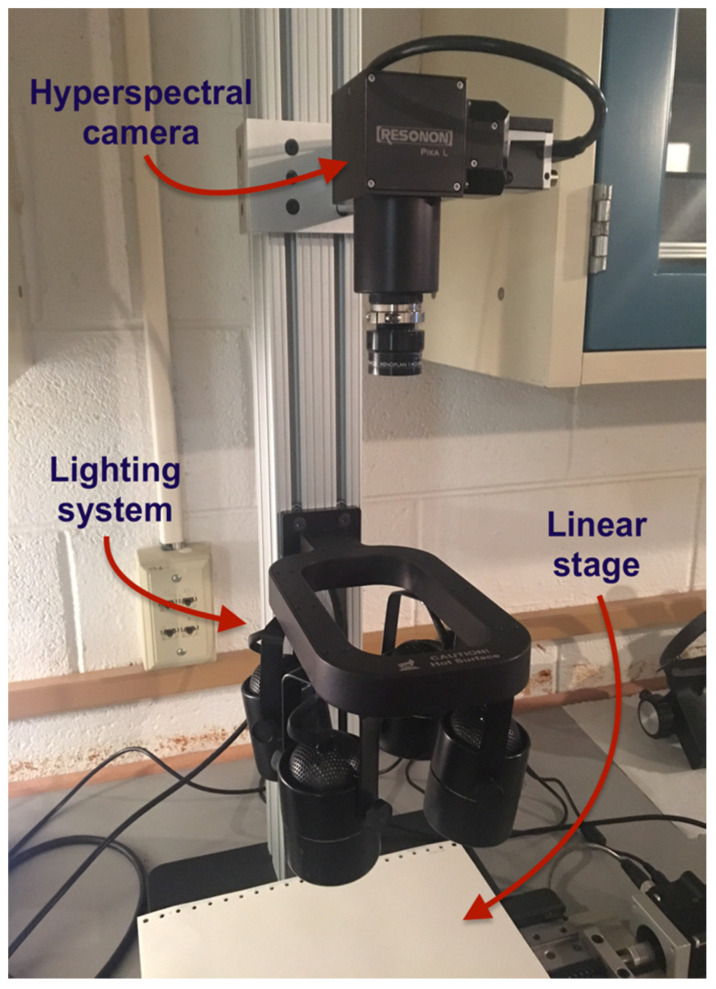
Hyperspectral camera setup, including halogen lighting system and linear stage.

**Figure 3:**
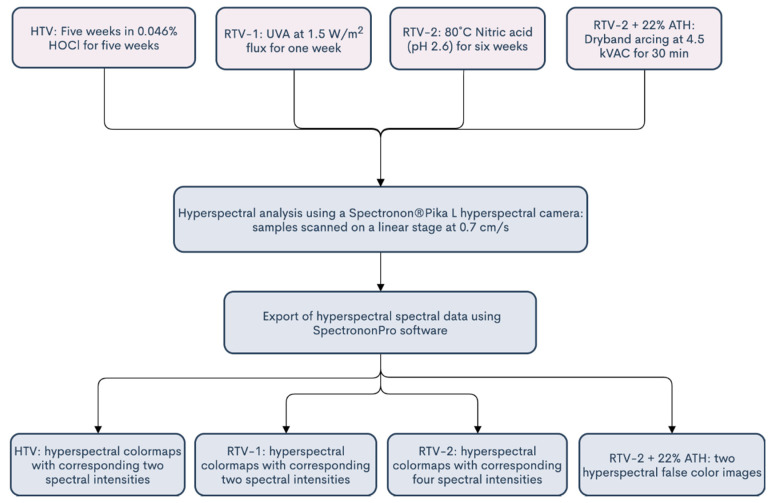
A flowchart encompassing the aging (light red) and hyperspectral imaging (HSI) analysis methods (light blue) utilized in this study.

After aging, each of the four types of samples were photographed using a Resonon Pika L hyperspectral desktop camera (Resonon Imaging Systems, Bozeman, MT, USA), which has a spectral range between approximately 370–1000 nm and a resolution of 2.1 nm, with 281 spectral channels, 900 spatial channels, and a max frame rate of 249 (Figure 2) [13]. The entire benchtop hyperspectral camera setup is shown in Figure 2. The setup consisted of a motorized linear scanning stage, four halogen lights, and the Pika L hyperspectral camera which was mounted on a track above (Figure 2). Halogen lights were utilized due to their ability to provide adequate illumination at all wavelengths [13]. The Pika L camera remained stationary, as the system utilizes a push-broom approach, where samples move relative to the camera. The linear stage was set to 0.7 cm/s. A flowchart of the entire process, including the aging methods and HSI analysis methods, is shown in Figure 3.

All images were taken using the Pika L camera, then processed and analyzed using the hyperspectral image analysis software SpectrononPro. As the Pika L can take both standard Red Green Blue (RGB) images, as well as hyperspectral datacubes, RGB images of all samples were first obtained using the hyperspectral camera and the SpectrononPro software. Afterwards, hyperspectral cubes, spectral colormaps, and wavelength signature plots were obtained for each specimen. For the purposes of this study, spectral wavelengths were plotted as a function of spectral intensity. For comparison, visual HSI colormaps were created using the average HSI spectral signatures for the overall samples as a function of wavelength and spectral radiance (intensity).

## 4. Results and Discussion

HSI imaging successfully determined changes and spectral differences in SIR materials after aging. Visual and spectral changes were observed using colormaps produced by the SpectrononPro software, which showed highlighted areas with different average wavelengths. Although there were notable differences among the different samples, there was a comparable change in spectral plots between aged and un-aged SIR material, regardless of the particular SIR formulation.

### 4.1. HTV

**Figure 4:**
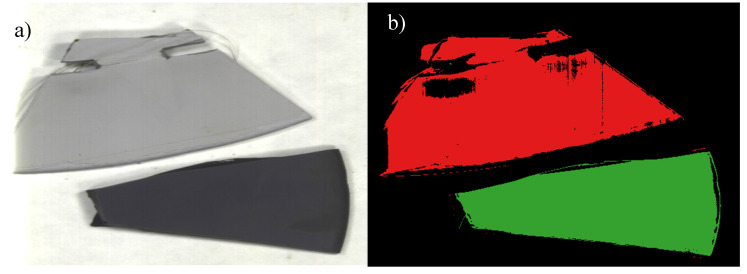
Red Green Blue (RGB) (**a**) and corresponding colormap image (**b**) for virgin (green) and aged (red) high temperature vulcanized (HTV).

Following HOCl aging, aged HTV was assessed next to a virgin un-aged sample for comparison. HSI software easily distinguished between aged and virgin RTV samples, as the two HTV samples exhibited significant spectral differences (Figure 4a,b). This was not entirely unexpected, as the aged sample was notably lighter in color compared to the virgin sample (Figure 4a), which was visible to the naked eye. Additionally, STRI hydrophobicity decreased from HC1 to HC7, and Shore A hardness decreased from 92 to 88 (Table 2) after aging, signifying that significant aging damage occurred both to the surface and to the bulk material [6]. Our previous research has indicated that HOCl can promote chain scission and the formation of voids and cavities within the material, resulting in a decrease in hardness over time [6], and this aging damage also results in a decrease in hydrophobicity [6]. Therefore, it may be possible that HSI analysis may be able to detect these changes, though additional verification studies will be necessary.

**Figure 5:**
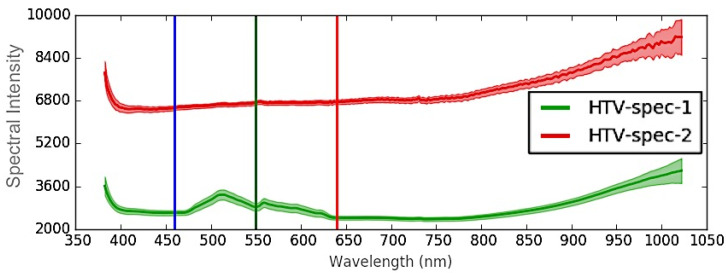
Wavelength plot for virgin (green) and aged (red) HTV.

Nevertheless, the spectral signature results indicated that the changes occurred primarily between 480–640 nm (Figure 5), and the spectral intensity was much lower for the virgin sample. Specifically, the virgin sample exhibited peaks in spectral intensity at 510 and 560 nm, while the aged sample lacked these peaks altogether (Figure 5). Apart from intensity, spectral patterns for both the virgin and aged HTV were similar above 700 nm.

### 4.2. RTV-1

**Figure 6:**
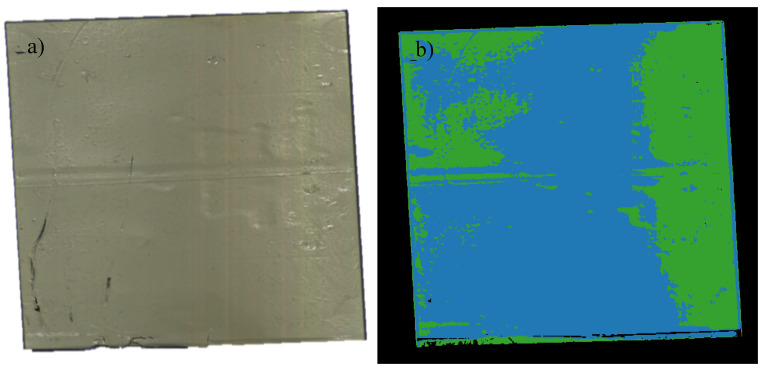
RGB (**a**) and corresponding colormap image (**b**) for UVA aged RTV-1 showing standard RTV-1 material (blue) and low molecular weight oil on the surface (green) HTV.

**Figure 7:**
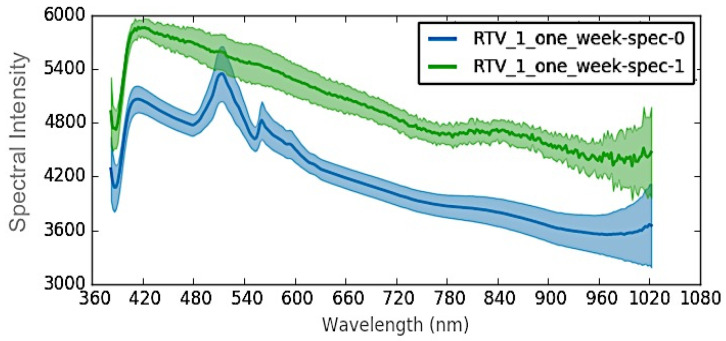
Wavelength plot for RTV-2 (blue) and RTV-2 low molecular weight oil (green).

After UVA aging for one week, the aged RTV-1 sample was assessed using HSI for differences in spectral signatures between the RTV-1 substrate and the LMW oil, which had developed on the surface. Although not highly visible in the RGB image (Figure 6a), a thin layer of LMW oil was visible to the naked eye on the RTV-1 surface following aging. STRI hydrophobicity for the RTV-1 sample decreased from HC1 to HC2 after aging, and Shore A hardness decreased from 21 to 17.5 (Table 2), signifying that some changes to the material did occur, and are likely due to the large losses of LMW oil. HSI was then used to assess differences in spectral signatures between the sample and the oil. As shown in Figure 6b, the HSI colormap indicated spectral differences between the RTV-1 substrate and the oil. Similar to the aged HTV sample, the spectral signature changes occurred primarily between 480–640 nm, as shown in Figure 7.

As with the HTV, spectral peaks were present at 510 and 560 nm for the RTV-1 substrate, at a lower spectral intensity, but were absent for the LMW oil. It may thus be hypothesized that the lack of peaks at 510 and 560 nm in the HTV may be correlated with a less crosslinked and thus more damaged material, as the LMW oil (green) on the RTV-1 lacks crosslinking and contains smaller and shorter polymer fragments. The damaged HTV material may similarity contain shorter, damaged fragments due to aging and chain scission, and thus record similarity when assessed using HSI. This is despite the fact that there were color differences between the materials, as the LMW oil was predominantly transparent and the aged HTV a lighter grey color, however both exhibited a loss in peaks after exposure to aging. By contrast, the RTV-1 standard bulk material (blue) recorded the spectral pattern peaks, similar to the unaged HTV, signifying that it detected unchanged material. This corresponds to our previous study [4], which showed that UVA does not induce chemical damage to the base RTV-1 material but does result in the production of LMW oil [4]. Nevertheless, both the LMW oil and aged HTV material appear to be recorded by the HS camera as lacking peaks at 510 and 560 nm, resulting in similar spectral pattern changes. It is possible that additional hyperspectral signatures may be required to identify these materials, and thus additional research will be required to properly identify and distinguish these differences.

### 4.3. RTV-2

**Figure 8:**
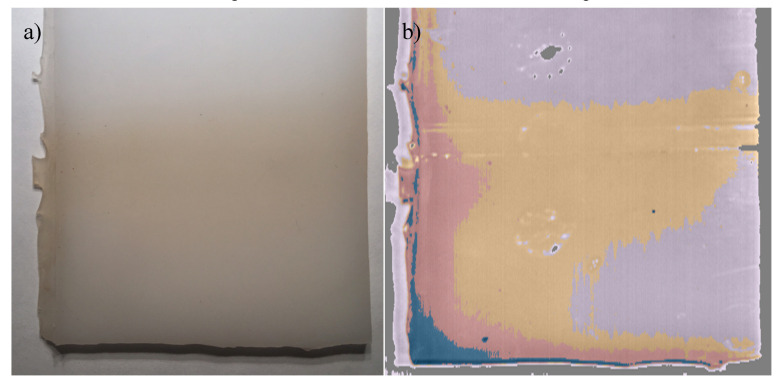
RGB image (**a**), and hyperspectral colormap image showing damaged areas not visible in RGB image (**b**)**.**

As with the RTV-1 and HTV samples, the RTV-2 sample aged for 6 weeks in nitric acid exhibited the same spectral signature changes between 480–640 nm, but with a few differences. Additionally, STRI hydrophobicity for the RTV-2 sample showed a decrease from HC1 to HC6 after aging, and Shore A hardness decreased from 23 to 12 (Table 2), signifying damage to the bulk material. While the RGB image (Figure 8a) showed some discoloration along the left edge of the sample, the HSI colormap was able to more precisely indicate difference in spectral changes (Figure 8b).

**Figure 9:**
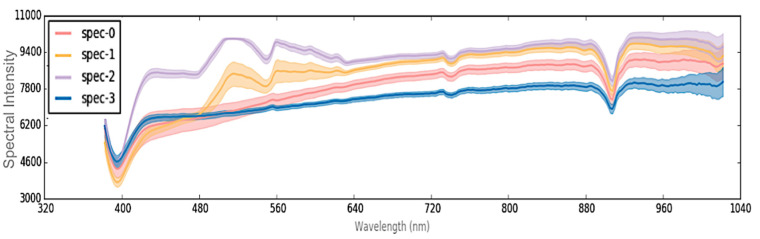
Wavelength plot for corresponding colormap image depicting varying levels of spectral radiance intensity for different areas of the RTV-2.

Different colors in the colormap image (Figure 9) (purple, orange, red, and blue) indicated different average spectral signatures, which the HSI software was able to detect. These colors correlated to the spectral signatures shown in Figure 8. While very similar spectral dips occurred at 730 and 900 nm for all colors/regions measured, there were significant spectral differences between 480–640 nm among the different colors/regions. The blue and red bands at the bottom left corner of the sample recorded no spectral peaks at 510 and 560 nm, unlike the larger purple area, which had noticeable spectral signature peaks at those wavelengths (Figure 9). The orange band, between the purple and red areas, recorded peaks at 510 and 560 nm, but at a lower spectral radiance.

It could be therefore conjectured that the presence of spectral peaks at 510 and 560 nm is associated with SIR materials, as it was evident in all the SIR samples, and the absence of these peaks could indicate damage to the underlying SIRs. This may be further supported by our previous work, which found that aging SIRs in nitric acid and HOCl can cause decreases in material hydrophobicity [4,5,6], and the formation of LMW material in solution, as a result of chain scission. If allowed to continue, however, this type of aging can ultimately affect the material’s electrical resistance over time, and silicone rubber insulators may have a decreased lifespan if hydrophobicity is diminished. Because the HS camera was able to detect spectral variations in the nitric acid aged sample, a possible correlation between spectral intensity of the spectral peaks could also be established to determine the level or type of aging of SIRs.

### 4.4. RTV-2 + 22% ATH

**Figure 10:**
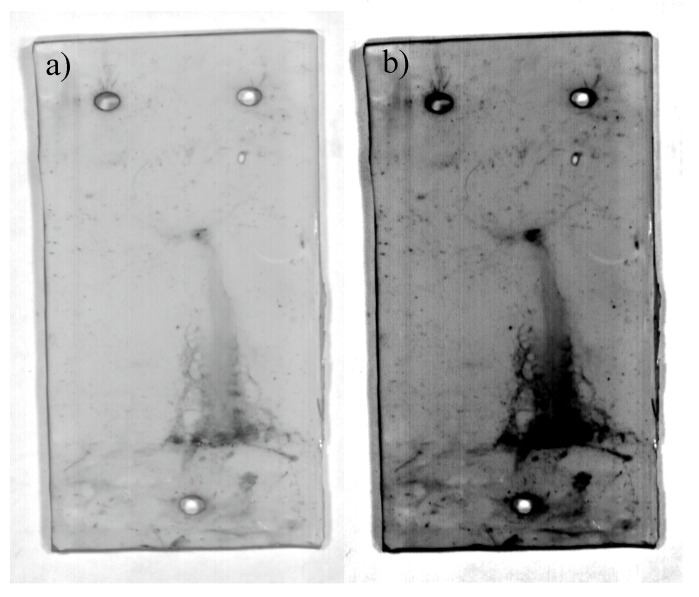
RTV-2 +22% ATH (aluminum trihydrate) sample after 30 min of dry-band arcing (**a**) and hyperspectral false color image showing damage (**b**).

**Figure 11:**
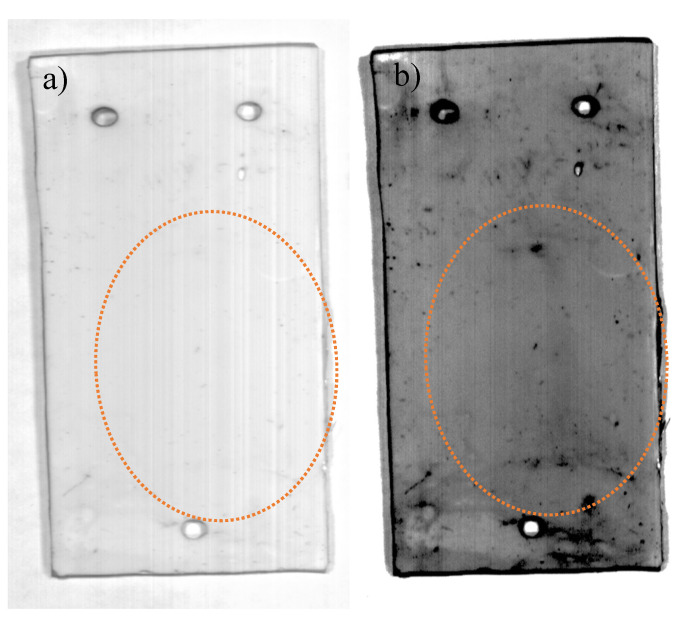
Sample in Figure 9 after being thoroughly cleaned with alcohol (**a**) and hyperspectral false color image (**b**).

The last sample, RTV-2 + 22% ATH, was assessed slightly differently than the previous SIR samples, as samples were assessed using greyscale and hyperspectral false color imaging. The RTV-2 + 22% ATH sample was first exposed to DBA for 30 min at 4.5 kVAC and then analyzed using HSI before and after the sample surface was cleaned using isopropyl alcohol. Surface damage was initially visible to the naked eye in the form of slight discoloration after DBA (Figure 10a) due to the tracking of ionic contaminate solution (Figure 10b) but was completely imperceptible after cleaning (Figure 11a). No notable changes in STRI hydrophobicity or hardness were recorded (Table 2) after initial dry band arcing. Nevertheless, changes in hyperspectral spectral signatures occurred between 400 and 650 nm; however, the areas of damage most visible in greyscale images occurred at band 428. Damage was also visible in the HSI false color image even after cleaning (Figure 11b), indicating that DBA did in fact cause permanent changes to the SIR material.

## 5. Conclusions

Our study indicates that hyperspectral imaging could be used to detect RTV damage, even if it is not readily visible to the human eye (Figure 11a,b) or in RGB images. The spectral signature changes occurred primarily between 400–650 nm (Figure 5, Figure 7 and Figure 9), and were consistent among the aged SIR samples regardless of formulation, color, or aging mechanism. Spectral intensity peaks were present at 510 and 560 nm for virgin or minimally aged SIR substrates, but were absent for aged samples and LMW oil, irrespective of SIR formulation. While FTIR analysis was attempted on the nitric acid aged sample, it did not show noticeable differences between the aged and unaged SIR portions, likely because some of the material changes were outside the range for FTIR analysis.

However, as this study is a proof of concept, it contains limitations and more analysis is still needed to discern the exact mechanisms of chemical damage seen in this study and expand upon the results to determine the best approach for assessing polymeric damage using HSI. For example, the small changes in hydrophobicity in the RTV-1 UVA aged sample may be due to the presence and scattering of LMW oil, and not necessarily reflect damage to the material itself. Furthermore, HSI may detect LMW oil as actual aged material, due to the lack of spectral peaks, rather than surface material. Therefore, more studies are needed to discern these differences in real case scenarios.

Nevertheless, there appears to be great potential for HSI to assess and detect damage in polymeric materials, such as SIRs, due to its ability to discern material changes. HSI could be used for remote sensing of polymer damage, pollution and contaminant accumulation, and LMW oil production in NCIs. Additionally, UAVs could be used to remotely analyze and capture HSI data from NCI insulators across a large area, and the data could then be used to correlate them to dry band arcing events or excessive corona arcing. Hyperspectral data, from either the field or the lab, could then be combined to assess the possibility of insulator failure while in-service. However, further investigations will be required to determine feasibility.

## Figures and Tables

**Table 1:** List of silicone rubbers (SIRs) and aging mechanisms investigated in this study.

Material	Aging Mechanism	Aging Time
HTV	0.046% Hypochlorous acid	5 weeks
RTV-1	UVA	1 week
RTV-2	80 °C Nitric acid (pH = 2.6)	6 weeks
RTV-2 + 22% ATH	Dry band arcing at 4.5 kVAC	30 min

**Table 2:** List of SIRs and aging mechanisms, as well as measured STRI hydrophobicity and Shore A hardness before and after aging.

Material	Aging Mechanism/Time	Measured STRI Hydrophobicity before Aging	Measured STRI Hydrophobicity after Aging	Measured Shore A Hardness before Aging	Measured Shore A Hardness after Aging
HTV	0.046% Hypochlorous acid for 5 weeks	HC 1	HC 7	92	88
RTV-1	UVA for 1 week	HC 1	HC 2	21	17.5
RTV-2	Nitric acid (pH 2.6) for 6 weeks	HC 1	HC 6	23	12
RTV-2 + 22% ATH	Dry band arcing at 4.5 kVAC for 30 min	HC 1	HC 1	34	34
